# Playing Music May Improve the Gait Pattern in Patients with Bilateral Caloric Areflexia Wearing a Cochlear Implant: Results from a Pilot Study

**DOI:** 10.3389/fneur.2017.00404

**Published:** 2017-08-17

**Authors:** Ann Hallemans, Griet Mertens, Paul Van de Heyning, Vincent Van Rompaey

**Affiliations:** ^1^Faculty of Medicine and Health Sciences, Department of Rehabilitation Sciences and Physiotherapy, University of Antwerp, Antwerp, Belgium; ^2^Multidisciplinary Motor Center Antwerp, University of Antwerp, Antwerp, Belgium; ^3^Faculty of Medicine and Health Sciences, Department of Translational Neurosciences, University of Antwerp, Antwerp, Belgium; ^4^Department of Otorhinolaryngology and Head & Neck Surgery, Antwerp University Hospital, Edegem, Belgium

**Keywords:** gait, profound hearing impairment, cochlear implant, auditory information, music

## Abstract

**Hypothesis:**

Auditory information through an active cochlear implant (CI) influences gait parameters in adults with bilateral caloric areflexia and profound sensorineural hearing loss.

**Background:**

Patients with bilateral caloric areflexia suffer from imbalance, resulting in an increased risk of falling. In case of simultaneous deafness, the lack of auditory feedback results in less awareness of the auditory scene. This combination might produce significant challenges while walking and navigating. Auditory cues can be restored to some extent with a CI. Electrical stimulation through a CI can also produce a vestibulocollic reflex through current spread, which can be measured as cervical vestibular-evoked myogenic potentials.

**Methods:**

Adults (seven males, one female, mean age 61 ± 14 years), wearing a CI to treat profound sensorineural hearing loss and presenting with bilateral caloric areflexia walked barefoot, over ground, at self-selected speed in three different conditions: with CI turned on, while listening to music and with CI turned off. Spatiotemporal and kinematic parameters of gait were calculated using the conventional gait model.

**Results:**

Removing auditory feedback by turning off the CI decreased stride time (mean difference 0.03 ± 0.15 s) and slightly increased stride length (mean difference 0.5 ± 1.2 cm) compared to the control condition with the CI on. Walking while playing music positively affected gait compared to walking with the CI on but without auditory feedback. By increasing the motion of the pelvis (mean difference 1.3° ± 0.4°), the knee (mean difference 3.9° ± 0.8°) and the ankle (mean difference 2.2° ± 0.2°), stride length increased (7.8 ± 1.2 cm), while stride time decreased (0.059 ± 0.016 s).

**Conclusion:**

Although a practice effect cannot be completely ruled out, this pilot study suggests that playing music while wearing an active CI may improve gait in patients with bilateral otovestibular loss. It remains unclear if the musical cues boost balance control or the CI might produce current spread and electrical stimulation to the vestibular afferents, thereby boosting its detection threshold, through stochastic resonance, and improving gait.

## Introduction

Hearing impairment is a major health concern in older adults, affecting more than 50% of adults aged 70 years and older (1, 2). Hearing impairment can have implications that go beyond poor communication. Because of the growing prevalence of hearing problems with increasing age, it is vitally important to study these implications (3). Several studies have shown that hearing loss has a major impact on mobility and activities of daily living, among which major walking difficulties such as a slow walking speed and reduced stride length (4).

Different hypotheses have been put forward to explain why hearing impairment might lead to poorer physical functioning during daily life, slower gait speed, and increased risk of falling. The most straightforward one is related to shared pathological pathways, as there are bilateral vestibular loss (2, 4, 5), increased cardiovascular risk (2, 4), or age-related brain dysfunction affecting both hearing and physical performance (6). Other hypotheses are related to decreased awareness of the auditory environment or competition of attentional resources (2, 4). These hypotheses not necessarily have to be mutually exclusive. Future research is needed to determine the relative importance of the underlying mechanistic pathways of the associations between hearing impairment and balance, gait and physical performance. Furthermore, it is important to investigate whether hearing loss treatments could influence these pathways (2, 5).

Given their common location in the inner ear, an impairment of the cochlea can also affect the vestibular sense organs, and thereby contribute to dizziness, impaired balance, and poorer physical functioning (2, 4). Thirty percent of people above 65 years of age experience some form of dizziness, increasing to 50% in persons above 85 years (7). In 4% of patients, dizziness can be explained by bilateral vestibular failure. And in about 25% of those patients, falls occur, leading to serious injuries and even death.

In this preliminary study, we aim to explore the relationship between cochlear implant (CI) stimulation, auditory feedback and gait in a specific patient population, i.e., adults with bilateral caloric areflexia and bilateral profound sensorineural hearing loss, wearing CIs. The goal of this study was to explore whether auditory sensory information mediated through the CI affects the spatiotemporal and kinematic parameters of gait in this population. We hypothesize that removal of auditory or electrical stimulation through the CI would negatively affect the gait pattern while auditory or electrical stimulation can have a positive effect on gait.

## Materials and Methods

### Study Design

A cross-sectional study was performed describing the gait pattern in three different conditions in a sample of hearing impaired adults wearing a CI and concomitant bilateral vestibular function loss.

### Setting

Participants were observed in a multidisciplinary, motion analysis laboratory, equipped with an automatic three-dimensional motion capture system [Vicon T10, 100 Hz., Vicon^®^ Oxford, UK, 100 fps, resolution 1 Megapixel (1,120 × 896)]. The conventional gait model marker setup (8) was used. After the anatomical calibration was successful, the subjects were instructed to walk barefoot, over ground, at self-selected speed in three different conditions: walking across with CI turned on (ON), walking across with CI turned on while listening to music at a comfortable sound level (M), and walking across with CI turned off (OFF). Music was played from two speakers located at the end of the walkway. Each condition was repeated three times. Conditions were performed in this order, without randomization. Subjects were allowed to rest in between trials to avoid fatigue. A safety harness was worn to prevent falls.

The study was performed according to the principles laid down in the Declaration of Helsinki; Recommendations guiding physicians in biomedical research involving human subjects. Adopted by the 18th World Medical Assembly, Helsinki, Finland, June 1964, amended by the 29th World Medical Assembly, Tokyo, Japan, October 1975, the 35th World Medical Assembly, Venice, Italy, October 1983, and the 41st World Medical Assembly, Hong Kong, September 1989. The study protocol was approved by the Committee for Medical Ethics UZA-UAntwerp (registration number B300201316328). The period of data collection was between September 2012 and April 2015.

### Participants

Participants were recruited from the Department of Otorhinolaryngology at the local university hospital. Eligibility criteria for participation were as follows: adults over 18 years of age diagnosed with a profound hearing impairment treated with a uni- or bilateral CI and diagnosed with bilateral caloric areflexia.

The hearing loss criteria for reimbursement of cochlear implantation were as follows: pure-tone average of 500, 1,000, and 2,000 Hz in unaided liminal audiometry exceeding 85 dB and speech discrimination with hearing aid less than 30%. All patients routinely underwent electronystagmography before cochlear implantation. Bilateral bithermal caloric testing was used to evaluate lateral semicircular canal function. The detailed methodology and normative values were reported earlier by Van der Stappen et al. (9).

Subjects were asked to provide date of birth and to report any musculoskeletal complaints over the last 6 months. Prior to participation, written informed consent was obtained. Patient anonymity was protected by using a unique identifier code in all experimental investigations.

### Variables of Interest

**Figure 1:**
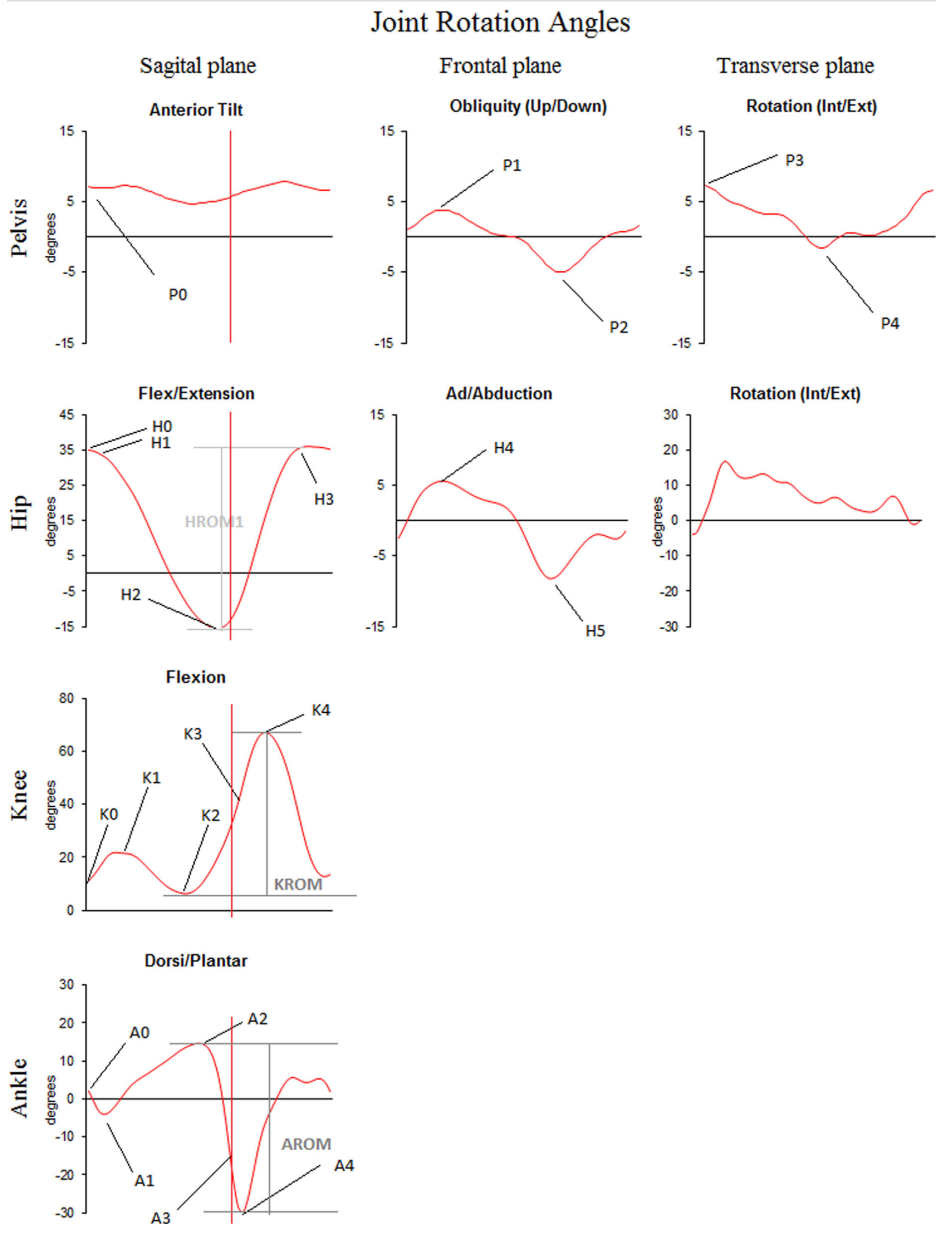
Graphical representation of the kinematic parameters of gait as selected from [Benedetti et al. (10)]—typical traces for joint rotation angles of the pelvis, hip, knee, and ankle in the sagittal, frontal, and transverse planes are plotted as a function of gait cycle duration. Traces represent the joint angular motion from foot strike to the next foot strike occurring with the same foot. Positive values indicate flexion, dorsiflexion, adduction, or internal rotation. Negative values indicate (hyper) extension, abduction, or external rotation. Variables of interest, as defined in Table 1, are indicated on the time traces.

Variables of interest were spatiotemporal and kinematic parameters of gait. An overview of each variable and the according definition are provided in Table 1 and Figure 1.

**Table 1:** Variables of interest and their definitions: spatiotemporal and kinematic parameters of gait are defined according to Benedetti et al. (10) and Hallemans et al. (11).

Variable	Units	Definition
Stride time	S	Time between foot strike and following foot strike with the same foot
Stride length	M	Longitudinal distance between foot strike and following foot strike with the same foot
Stance	%	Duration of stride time that the foot is on the ground
Step width	M	Mediolateral distance between left and right foot during double support
P0	Degree (°)	Anterior pelvic tilt, inclination of the pelvis at foot strike (sagittal plane)
P1	Degree (°)	Pelvis up, maximum angle of the pelvis in stance (frontal plane)
P2	Degree (°)	Pelvis down, minimum angle of the pelvis in swing (frontal plane)
P3	Degree (°)	Internal rotation, maximum angle of the pelvis in stance (transverse plane)
P4	Degree (°)	External rotation, minimum angle of the pelvis in swing (transverse plane)
H0	Degree (°)	Hip flexion angle at foot strike (sagittal plane)
H1	Degree (°)	Hip flexion in stance, maximum hip angle in stance (sagittal plane)
H2	Degree (°)	Hip extension, minimum hip angle in stance (sagittal plane)
H3	Degree (°)	Hip flexion in swing, maximum hip angle in swing (sagittal plane)
HROM1	Degree (°)	Hip flexion—extension range of motion
H4	Degree (°)	Hip adduction in stance, maximum hip angle in stance (frontal plane)
H5	Degree (°)	Hip abduction in swing, minimum hip angle in swing (frontal plane)
K0	Degree (°)	Knee flexion angle at foot strike (sagittal plane)
K1	Degree (°)	Shock—absorbing knee flexion in stance (sagittal plane)
K2	Degree (°)	Knee extension in stance, minimum angle in stance (sagittal plane)
K3	Degree (°)	Knee flexion angle at toe off (sagittal plane)
K4	Degree (°)	Max knee flexion in swing (sagittal plane)
KROM	Degree (°)	Knee flexion—extension range of motion
A0	Degree (°)	Ankle angle at foot strike (sagittal plane)
A1	Degree (°)	Ankle plantar flexion during weight acceptance, minimum angle in stance (sagittal plane)
A2	Degree (°)	Peak ankle dorsiflexion, maximum angle in stance (sagittal plane)
A3	Degree (°)	Ankle plantar flexion angle at toe off (sagittal plane)
A4	Degree (°)	Peak ankle plantar flexion after push-off, minimum angle in swing (sagittal plane)
AROM	Degree (°)	Ankle plantar flexion—dorsiflexion range of motion

### Measurements and Data Calculations

For each subject, information was obtained on body mass, height, leg length, and width of the knee and ankle. All measures were taken according to standard procedures (8).

Reflective markers were tracked and labeled using the Vicon Nexus 1.8.5 software. Trajectories were filtered (low pass zero phase shift Butterworth filter, cutoff frequency 6 Hz.) and the conventional gait model was applied to calculate joint kinematics (8, 12). Standard Euler/Cardan rotations of pelvis, hip, knee, and ankle were considered. For the knee and ankle, focus was on the joint angular motions in the sagittal plane (flexion and extension). For the pelvis and the hip, movements in the frontal and transverse plane were also considered. Based on visual inspection of the ankle marker (*malleolus lateralis*) trajectories, instances of foot strike and foot off were determined.

When all markers were visible for at least three consecutive strides, trials were further processed. The c3d files obtained in Vicon Nexus 1.8.5 were exported to a custom-made MATLAB (R2015a for Windows) model to calculate the variables of interest. Stride time, stance duration, stride length, and step width were calculated based on the left and right ankle marker trajectories. Outcome parameters (described in Table 1) were determined on the three-dimensional pelvis, hip, knee, and ankle according to Figure 1.

### Bias

Possible sources of bias include selection bias. Subjects participated on a voluntary basis. Those who have severe impairments due to their condition might be less likely to participate.

### Quantitative Variables

Spatiotemporal and kinematic parameters of interest were calculated for each stride in each trial. Prior to further data analysis median values for each parameter were calculated from the different strides in a trial.

### Statistical Analysis

We ran a mixed-model analysis of variance (ANOVA) model using the mixed procedure in SPSS 23.0 (SPSS, Inc. Chicago, IL, USA). The model controlled for the within-subject nature of the three conditions (ON, M, and OFF) by including random effects for participant, with a variance components covariance structure and restricted maximum likelihood estimation. Trial number was included as a covariate, between-subjects factor to model a possible practice effect, but there were no significant main effects [*F*(1, 99.214) = 0.089, *p* = 0.766]. Consequently, the trial number factor was dropped from the model. *Post hoc* pairwise comparisons, using Bonferroni corrections to reduce type I error, were performed to compare the three conditions to each other.

Statistical significance was set at *p* < 0.05 in all tests. Since no side differences were observed, left and right side data were pooled.

## Results

### Participants and Descriptive Data

Eight patients fitting the selection criteria were contacted to participate in this study. All agreed to participate and were included in the study. Descriptive data for the sample are presented in Table 2. Age ranges from 36 to 79 years and there is also a large spread in stature, mass, and body mass index.

**Table 2:** Descriptive characteristics of the participants in the study sample (P, patient number; M, male; F, female; CI, cochlear implant; BMI, body mass index).

P	M/F	Age (years)	CI side	Date of implantation	Implant type	Electrode type	Processor type	Height (mm)	Mass (kg)	BMI
1	M	68	R	8/03/2013	Concerto	Flex 28	Opus 2	1800	92.7	28.6
2	M	79	R	9/02/2000	CI24M		CP810	1740	74.3	24.5
3	M	65	L	25/06/2014	Concerto PIN	Flex 28	Opus 2 + RONDO	1680	94.6	33.5
4	F	59	R	16/04/2013	Concerto PIN	Flex 28	Opus 2	1595	90	35.4
5	M	49	R	10/07/2012	Concerto PIN	Flex 28	Opus 2	1760	81.8	26.4
6	M	54	R	25/07/2006	Pulsar		Opus 2	1705	114	39.2
7	M	36	L + R	08/06/2011 + 15/04/2011	Sonata ti100 Concerto ti100		Opus 2	1775	77.3	24.5
8	M	77	R	11/04/2006	Pulsar 100	Flex	Opus 2	1640	92.2	34.3

### Outcome Data

For each participant, in each of the three conditions, up to three trials were recorded. In each trial between three and six strides were obtained with good visibility of all markers. For each variable of interest, the median value over the different number of strides was calculated prior to further analysis. Not all participants were able to complete all three trials in each condition. Reasons for not being able to analyze a trial were poor marker visibility or artifacts in the data due to soft tissue movements. These usually only occurred in one out of three trials so, there were always two remaining trials for analysis. In the ON condition, 20 out of 22 trials were suitable for analysis, 18 out of 22 in the M condition, and 19 out of 22 in the OFF condition. Missing data were treated as missing.

### Main Results

**Figure 2:**
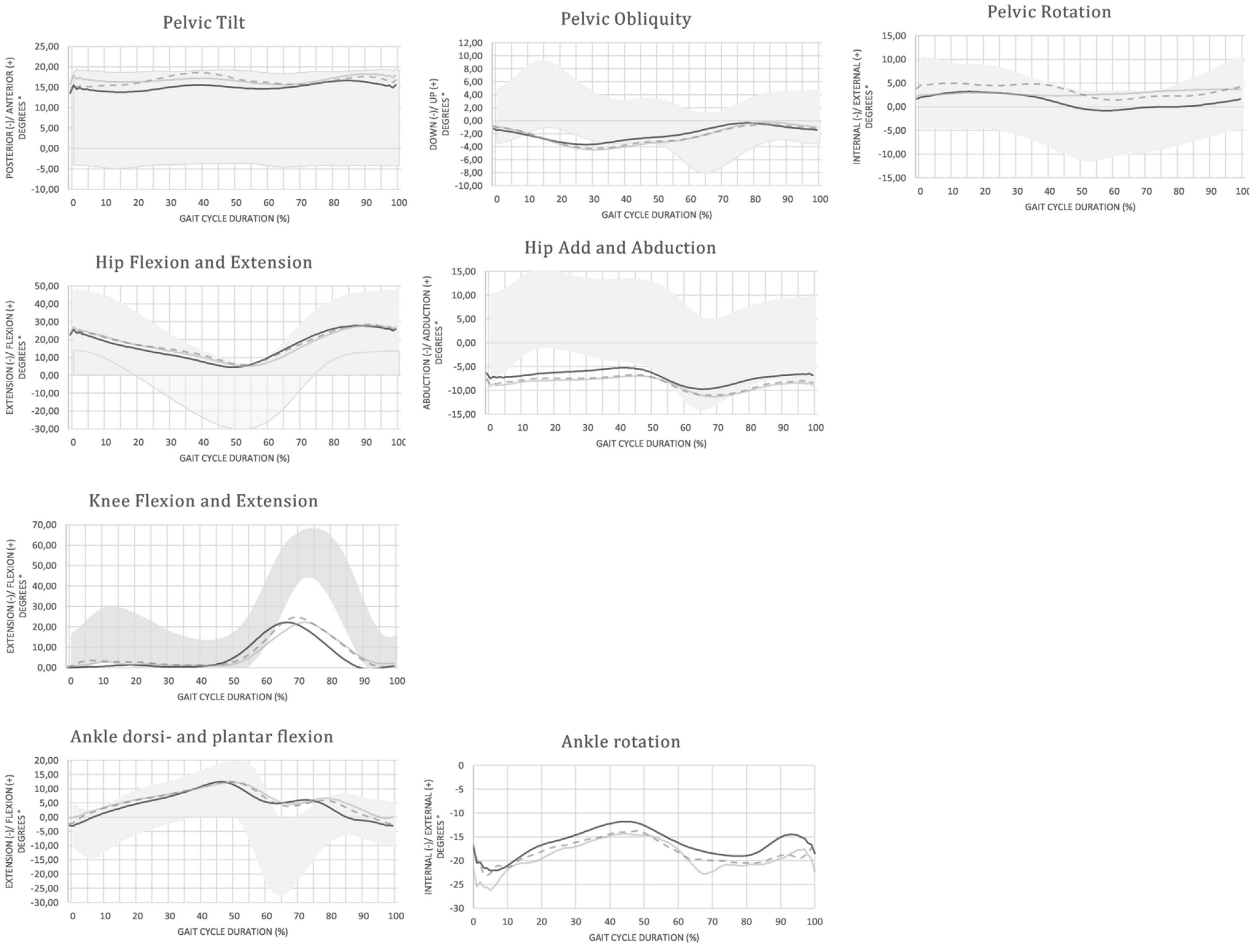
Joint angular time profiles of one representative individual in different conditions: ON (black solid line), OFF (gray solid line), and M (gray dashed line) compared no normative values (gray band)—the patient with vestibular loss shows a limited range of motion in all joints compared to the normative values. The more dynamic and functional gait pattern in the M condition is revealed by the increased range of motion in the sagittal plane.

An overview of the mean (±SD) spatiotemporal and kinematic parameters in each condition is provided in Table 3. Joint angular time profiles of one representative individual are demonstrated in different conditions in Figure 2.

**Table 3:** Mean and SD of the step-time and kinematic parameters when walking with the CI turned on (ON), walking with the CI off (OFF), and walking while listening to music (M).

Variable	Units	ON mean	SD	OFF mean	SD	M mean	SD	Reference range
Stride time*	s	1.11	0.11	1.08	0.13	1.05	0.10	0.92–1.04
Stride length*	m	0.92	0.20	0.95	0.22	0.99	0.19	1.04–1.35
Stance	%	66.8	2.20	67.7	1.91	67.4	2.27	60.1–66.8
Step width	m	0.24	0.05	0.24	0.06	0.23	0.11	0.22–0.27
P0	Degree (°)	16.2	5.3	13.5	5.9	14.6	5.7	–
P1	Degree (°)	1.2	1.9	1.8	4.8	2.5	4.7	–
P2	Degree (°)	−2.1	1.6	−2.4	1.9	−2.8	1.9	–
P3*	Degree (°)	4.4	3.6	3.7	4.4	4.0	5.0	–
P4	Degree (°)	−2.8	3.1	−3.1	3.1	−3.4	4.3	–
H0	Degree (°)	34.5	7.8	32.5	7.9	33.9	7.3	–
H1	Degree (°)	34.5	7.8	32.5	7.8	34.1	7.2	–
H2	Degree (°)	5.1	7.6	2.8	7.0	2.0	7.2	–
H3	Degree (°)	35.9	7.6	34.4	7.0	36.0	6.4	–
HROM1	Degree (°)	31.1	5.7	31.6	6.1	34.0	5.5	50.1–59.2
H4	Degree (°)	1.9	5	1.5	6.4	3.2	7.5	–
H5	Degree (°)	−6.5	5.2	−8.0	4.7	−7.8	5.0	–
K0	Degree (°)	4.9	5.3	6.8	7.3	6.1	6.4	–
K1	Degree (°)	28.9	13	35.5	14.6	36.8	16.7	–
K2	Degree (°)	1.7	5.3	4.2	8.0	2.5	7.2	–
K3*	Degree (°)	28.9	13	35.7	14.5	36.8	16.7	–
K4	Degree (°)	30.9	12	38.0	14.0	39.7	16.1	–
KROM*	Degree (°)	29.4	11	33.7	10.4	36.9	13.8	39.7–43.1
A0	Degree (°)	−3.8	3.5	−2.4	3.9	−2.8	4.0	–
A1	Degree (°)	−6	3.4	−6.5	5.1	−8.5	4.1	–
A2	Degree (°)	14.3	5.7	14.8	5.4	14.2	4.6	–
A3*	Degree (°)	−1.3	5.3	−3.3	5.8	−5.0	4.9	–
A4	Degree (°)	2	3.5	3.0	2.6	2.7	2.3	–
AROM	Degree (°)	12.2	4.1	11.8	4.3	11.4	3.5	–

*Reference values are provided based upon literature data (10)*.

*A significant main effect of condition (*p* < 0.05).

In the mixed model ANOVA, a significant main effect of condition was found on stride time [*F*(2, 100.035) = 21.345, *p* < 0.001] and stride length [*F*(2, 100.015) = 23.312, *p* < 0.001]. No significant effect of condition was found on stance duration [*F*(2, 99.541) = 0.156, *p* = 0.881] or step width [*F*(2, 100.069) = 0.342, *p* = 0.711].

Regarding the kinematic parameters, the mixed model ANOVA showed a significant main effect of condition on P3 [*F*(2, 39.064) = 4.819, *p* = 0.013], K3 [*F*(2, 39.016) = 5.853, *p* = 0.006], K4 [*F*(2, 39.016) = 9.588, *p* < 0.001], KROM [*F*(2, 39.009) = 8.252, *p* = 0.001], and A3 [*F*(2, 39.042) = 7.429, *p* = 0.002].

### *Post Hoc* Pairwise Comparisons

Table 4 represents the results from the *post hoc* pairwise comparisons. Stride time is significantly shorter in OFF (mean = 1.073 s, SD = 0.041 s) and M (mean = 1.060 s, SD = 0.041 s) compared to ON (mean = 1.120 s, SD = 0.041 s) with a mean difference of 0.047 s [(0.024 s, 0.070 s), *p* < 0.001] and 0.059 s [(0.036 s, 0.083 s), *p* < 0.001], respectively. Stride length in M (mean = 99.7 cm, SD = 7.2 cm) increases compared to ON (mean = 91.9 cm, SD = 7.2 cm); mean difference 7.8 cm (5.0 cm, 10.6 cm), *p* < 0.001, and stride length in OFF (mean = 96.5 cm, SD = 7.2 cm) significantly increases; mean difference 4.6 cm (1.9 cm, 7.4 cm), *p* < 0.001. Stride length in M is also significantly larger compared to OFF [mean difference 3.1 cm (0.3 cm, 6.0 cm), *p* = 0.028].

**Table 4:** Results from the *post hoc* pairwise comparisons.

Variable	Units	OFF–ON mean	SD	*p*	Power	M–ON mean	SD	*p*	Power
Stride time	s	−0.055	0.003	<0.001	1.000	−0.065	0.003	<0.001	1.000
Stride length	m	0.045	0.005	<0.001	1.000	0.071	0.005	<0.001	1.000
P3	Degree (°)	–	–	–	–	1.7	0.4	0.016	0.941
K3	Degree (°)	–	–	–	–	3.2	0.8	0.022	0.911
KROM	Degree (°)	–	–	–	–	3.8	0.9	0.020	0.920
A3	Degree (°)	–	–	–	–	−2.6	0.2	<0.001	1.000

*Effect sizes (mean and SDs), level of significance, and power comparing OFF to ON and comparing M to ON are presented*.

For kinematic parameters, differences occur between M and OFF. Walking while listening to music leads to an increase in internal pelvic rotation [P3 mean difference 1.3° (0.06°, 2.5°), *p* = 0.037], an increase in the knee flexion angle at toe off [K3 mean difference 3.0° (0.8°, 5.2°), *p* = 0.022], an increase in knee flexion and extension range of motion [KROM mean difference 3.9° (1.5°, 6.3°), *p* = 0.001], and an increase in ankle plantar flexion at toe off [A3 mean difference 2.2° (0.7°, 3.6°), *p* = 0.002].

## Discussion

A total body gait analysis was obtained for eight patients with bilateral caloric areflexia wearing a CI because of profound bilateral sensorineural hearing loss. Turning off the CI influenced the gait pattern: stride time decreased and stride length slightly increased but no effects were found on the kinematics of gait. These findings reject the hypothesis that removal of auditory or electrical stimulation through the CI negatively affects the gait pattern. Since conditions were not randomized, a practice effect might be an attributable factor. Nevertheless, a significant main effect of playing music when turning the CI on was found on the spatiotemporal and the kinematic parameters of gait, on top of the possible practice effect. By increasing the motion of the pelvis, the knee, and the ankle, stride length increased. Stride time decreased leading to an increase in stride frequency. Together these changes in gait led to a more efficient walking pattern. These results suggest that addition of auditory cues through an active CI may improve gait in patients with bilateral caloric areflexia, although further research with sound methodology is necessary.

As far as the authors are aware this study is the first to report on gait characteristics in a population of adults with profound hearing impairment, treated with a CI and diagnosed with bilateral caloric areflexia. The benefit of selecting this specific population is that it allows identification of the impact of vestibular loss on gait parameters. Also, the effects of treating hearing impairment with a CI can be investigated. Previous studies have considered the effect of CI on postural (standing) balance and have found improvements in postural control (13–16) after implantation, especially in more demanding situations. Explanatory hypothesis formulated are related to either increased possibility for vestibular compensations after CI implantation or improved awareness of the environment through auditory feedback. In a recent paper, Parkes and coworkers (17) showed that electrical stimulation from a CI could elicit VEMP responses. This would confirm spread of current to the vestibular system. Theoretically, this phenomenon may provide a usable vestibular cue, background information that may boost weak but intact residual vestibular function and thereby enhance central signal processing, a mechanism called stochastic resonance (17). Previous studies used this principle to enhance the sensitivity of residual vestibular afferents in patients with bilateral caloric areflexia by using galvanic vestibular stimulation (GVS), which involves electrical stimulation vestibular afferents. Thereby, imperceptible amounts of noisy GVS (nGVS) were shown to improve posture in healthy individuals while walking under visual deprivation and posture and walking performance in BVF patients (18–21). The results of previous studies on postural control (13–16) and vestibular function (14, 17) are in line with the observations in this study that turning the CI on and stimulating the CI lead to a more efficient gait pattern.

Despite the innovative character of the study, several limitations need to be considered. By only including people with vestibular loss, the hypothesis regarding shared pathological pathways between hearing impairment and vestibular loss as an explanation for the increased risk of poor physical performance, reduced mobility, and increased risk of falling cannot be investigated. Due to the low prevalence of bilateral caloric areflexia, the sample size of this study is small. Furthermore, this was a sample of convenience so selection bias cannot be excluded. Despite the small sample size, significant effects were identified indicating the sample had sufficient power.

**Figure 3:**
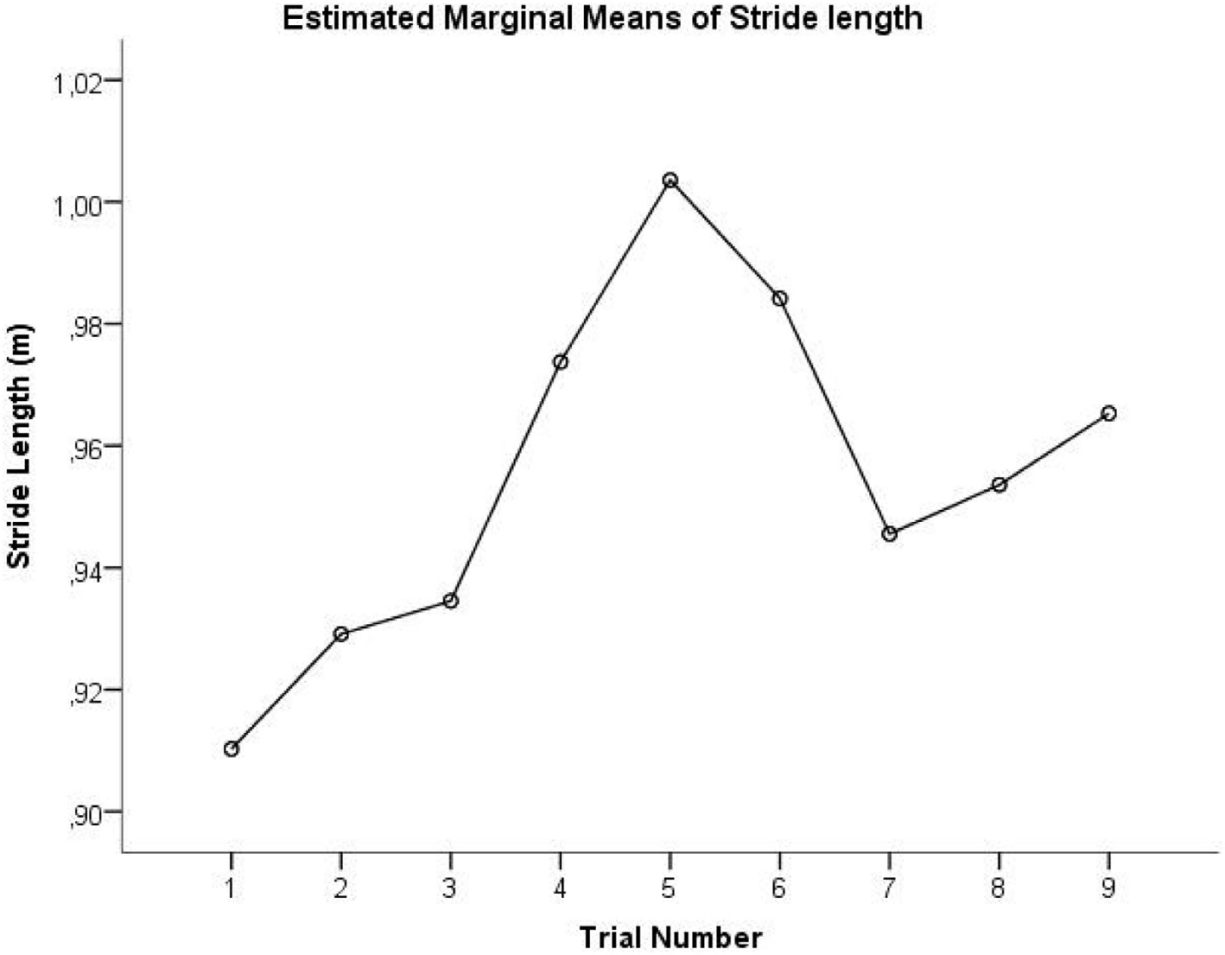
Estimated mean of stride length is plotted as a function of trial number. Trials 1–3 represent ON condition, trials 4–6 represent M condition, and trials 7–9 represent OFF condition.

A second limitation is that the different conditions were not randomized. Fatigue effects should not be an issue confounding the comparison between the three conditions since all subjects could rest in between the trials and nobody reported fatigue at the end of the measurement session. However, a practice effect cannot be ruled out, since the results obtained in the OFF condition are counterintuitive. This condition was performed last, when participants were probably more comfortable than in the first trials of the ON condition where participants might be stiff, nervous, or anxious. Using the linear mixed model we tried to account for this by adding trial number as a covariate. No significant main effect of trial number was observed. A possible explanation might be that the relation between gait performance and trial number in this specific study is not linear. Figure 3 shows the relation between stride length (estimated means from the linear mixed model) and trial number, suggesting a practice effect might indeed take place. However, stride length largely increases in trials 4–6, which represent the M condition. This suggests that playing music has an effect on gait, on top of the practice effect.

Overall, when the gait pattern of adults wearing a CI and presenting with vestibular loss is compared to that of healthy older individuals described in the literature (10), several differences are observed (Table 3). In all conditions, stride length is relatively small and stride duration is increased. Important kinematic differences are the small range of motion in the hip, knee, and ankle that are related to limited hip extension in stance, reduced knee flexion in swing, and reduced ankle plantar flexion at push-off. Similar adaptations in gait have been observed in other situations/populations, such as individuals with severe visual impairment and blindness, and have been related to lack of appropriate balance control strategies (22–25). Alternatively, reduced availability of cognitive resources might also explain (part of) the differences in gait that were observed. In older adults, early gait speed decline has been found a precursor to cognitive decline (26). In this study’s population, the attention required for processing of degraded sound signals might place a load on the listener’s cognitive resources. Turning the CI on might free these resources for other purpose such as control of balance and locomotion.

Altogether, sound seems an important source of information during gait, next to visual, vestibular and proprioceptive information. Turning on the CI and playing music positively affect gait, although the effect sizes are rather small. These results favor the hypothesis that impaired hearing might make moving more uncertain by disturbing perception of the environment since acoustic cues assist in perception of the environment while moving (27). This hypothesis is further supported by the observation that providing additional auditory cues (by playing music) improves gait mobility. Also, in healthy older adults, music introduced a significant increase in gait velocity mostly due to an increase in stride length (28). This is an important finding related to the design of rehabilitation programs that aim at improving mobility in older adults with hearing impairment (and vestibular loss). Furthermore, the results of this study might also favor the hypothesis that treatment of hearing impairment (e.g., through CI) may improve mobility problems as well. However, these results are very explorative and preliminary and should be further investigated in larger controlled studies. They contradict the finding of Kamil and coworkers (2) that hearing aid use did not affect mobility. A possible explanation might be related to the severity of hearing loss, which is much greater in people treated with CI than in those that receive a hearing aid. Consequently, the benefits of improving hearing might also be much greater.

To conclude, this pilot study demonstrates that addition of auditory cues through active CIs may improve gait in patients with bilateral otovestibular loss. It remains unclear if the auditory cues boost balance control or the CI might produce current spread and electrical stimulation to the vestibular afferents, or both, thereby boosting its detection threshold, through stochastic resonance and improving gait. Also, the use of music as a means of providing auditory cues to improve mobility should be further explored.

## Ethics Statement

This study was carried out in accordance with the recommendations of the Committee for Medical Ethics UZA-UAntwerp with written informed consent from all subjects. All subjects gave written informed consent in accordance with the Declaration of Helsinki. The protocol was approved by the Committee for Medical Ethics UZA-UAntwerp (registration number B300201316328).

## Author Contributions

All the authors had substantial contributions to the conception or design of the work (AH, VV, and PV); or the acquisition, analysis, or interpretation of data for the work (GM, AH, PV, and VV); all were involved in drafting the work (AH and GM) or revising it critically for important intellectual content (PV and VV) and provided final approval of the version to be published. All the authors agreed to be accountable for all aspects of the work in ensuring that questions related to the accuracy or integrity of any part of the work are appropriately investigated and resolved.

## Conflict of Interest Statement

The authors declare that the research was conducted in the absence of any commercial or financial relationships that could be construed as a potential conflict of interest.
